# Scalable Asymmetric Fabric Evaporator for Solar Desalination and Thermoelectricity Generation

**DOI:** 10.1002/advs.202406474

**Published:** 2024-09-20

**Authors:** Zhuan Fu, Dandan Zhong, Sijie Zhou, Leyan Zhang, Weihao Long, Jiajing Zhang, Xinyu Wang, Jiahao Xu, Jieyao Qin, Junyao Gong, Li Li, Liangjun Xia, Bin Yu, Weilin Xu

**Affiliations:** ^1^ State Key Laboratory of New Textile Materials and Advanced Processing Technologies Wuhan Textile University Wuhan 430200 P. R. China; ^2^ College of Textile Science and Engineering Zhejiang Sci‐Tech University Hangzhou 310018 P. R. China; ^3^ College of Textiles Donghua University Shanghai 201620 P. R. China; ^4^ School of Mechanical and Power Engineering East China University of Science and Technology Shanghai 200237 P. R. China; ^5^ School of Engineering The Hong Kong University of Science and Technology Hong Kong 999077 P. R. China

**Keywords:** asymmetric fabrics, basalt fibers, carbon black, interface evaporation, thermoelectricity

## Abstract

The integration of solar interfacial evaporation and power generation offers a sustainable solution to address water and electricity scarcity. Although water‐power cogeneration schemes are proposed, the existing schemes lack scalability, flexibility, convenience, and stability. These limitations severely limit their future industrial applications. In this study, we prepared a hybrid fabric composed of basalt fibers and cotton yarns with asymmetric structure using textile weaving technology. The cotton yarn in lower layer of fabric facilitates water transport, while the basalt fibers in upper layer enable thermal localization and water supply balancing. The carbon black is deposited on top layer by flame burning to facilitate photothermal conversion. The fabric exhibits a high evaporation rate of 1.52 kg m^−2^ h^−1^, which is 3.6 times that of pure water, and an efficiency of 88.06% under 1 kW m^−2^ light intensity. After assembly with a thermoelectric module, the hybrid system achieves a maximum output power density of 66.73 mW m^−2^. By exploiting the scalability of fabric, large‐scale desalination and power production can be achieved in outdoor environments. This study demonstrates the seamless integration of fabric‐based solar evaporation and waste heat‐to‐energy technologies, thereby providing new avenues for the development of scalable and stable water‐power cogeneration systems.

## Graphical abstract

A carbon‐deposited hybrid fabric is prepared using textile weaving and flame burning for solar evaporation. The design establishes an asymmetric carbon‐basalt‐cotton structure with photothermal conversion, thermal insulation, and water supply balancing. The further integration of thermoelectric generator modules to optimize waste heat utilization, presents a promising strategy for the development of stable and scalable water‐power cogeneration systems.

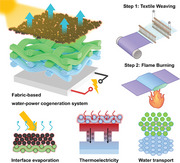

## 1 Introduction

Fresh water and electricity are intertwined requirements for modern societies.^[^ ^1^, ^2^ ^]^ Continuous global population growth and industrial and agricultural development have led to a constant increase in the demand for freshwater.^[^ ^3^, ^4^, ^5^ ^]^ The demand for environmentally sustainable and clean energy to mitigate climate change and reduce dependence on finite natural resources has also increased.^[^ ^6^, ^7^ ^]^ To address these challenges, it is crucial that integrated solutions for sustainable development be developed.

As an inexhaustible and environmentally friendly energy source, solar energy has attracted considerable attention for the production of green electricity and clean water.^[^ ^8^, ^9^ ^]^ Photothermal interfacial evaporation is an emerging freshwater production technology in which solar energy is utilized to heat liquids at the water–air interface through a thermo‐localization strategy aimed at generating steam efficiently.^[^ ^10^, ^11^ ^]^ Numerous effective photothermal conversion materials, such as carbon‐based materials,^[^ ^12^ ^]^ polymers,^[^ ^13^ ^]^ metal nanoparticles,^[^ ^14^ ^]^ and semiconductors^[^ ^15^ ^]^ have been reported. These materials have numerous applications when coupled with different carriers, such as thin films,^[^ ^16^ ^]^ aerogels,^[^ ^17^ ^]^ hydrogels,^[^ ^18^ ^]^ fabrics,^[^ ^19^, ^20^ ^]^ and biomass^[^ ^21^ ^]^ in which the solar vapor generation rate can be controlled by adjusting the type of photothermal material and carrier structure.

Among the various carriers, fabrics (e.g., woven,^[^ ^22^ ^]^ knitted,^[^ ^23^ ^]^ and nonwoven^[^ ^24^ ^]^) have significant advantages for solar interfacial evaporation due to their characteristics. These characteristics include: i) the multiscale structure of fibers and fabrics, which increases their light absorption capacity; ii) their high porosity and large specific surface area, which provide a large evaporation area and more vapor escape paths; iii) the availability of mature processing techniques for arbitrary structural design and size expansion; iv) dimensional stability and durability, which are conducive to various complex practical environments; and v) low cost and suitability for mass production. However, the lack of consideration for excess water transport in previous fabric designs leads to heat loss in the absorber and limited evaporation rates.^[^ ^25^, ^26^, ^27^ ^]^ In addition, the thinness of the fabric impedes effective thermal localization.^[^ ^10^ ^]^ Thermal localization and water supply balance in fabric‐based photothermal evaporators are fundamental bottlenecks to further improvements in evaporation performance. The diversity and processability of textiles offer promising avenues for overcoming the challenges mentioned. Properly designing fiber types and fabric structures can be an effective approach.^[^ ^28^, ^29^, ^30^, ^31^ ^]^ Among various fiber types, basalt fiber stands out as an inorganic high‐performance fiber, produced by melting and drawing natural basalt rock into continuous fibers. Its low thermal conductivity and moisture absorption make it an ideal candidate for addressing thermal positioning and water supply balance. Moreover, the superior mechanical properties, chemical stability, and heat resistance (ranging from −265 to 800 °C) of basalt fibers ensure excellent processability, weather resistance, and stability for fabric evaporators.^[^ ^32^ ^]^ Furthermore, waste heat is unavoidably generated during evaporation. Such low‐grade heat is usually neglected or directly absorbed by bulk water, resulting in wasted thermal energy.^[^ ^8^, ^33^ ^]^ This heat can be harnessed for temperature difference power generation to maximize the system energy utilization efficiency.

In this study, a hybrid fabric with a double‐layered structure was prepared using a textile weaving technique for solar‐driven water vapor generation. The upper and lower layers of the fabric were woven independently from basalt fibers and cotton yarns, respectively. The carbon black attached to the surface by flame burning acts as a photothermal conversion material for solar energy absorption and photothermal conversion. The basalt fibers in the upper layer of the fabric facilitate thermal localization at the top layer owing to their good thermal insulation, while the cotton yarns in the lower layer of the fabric provide a water transportation path to ensure adequate water supply. Water transfer from the cotton yarns to the contacting basalt fibers via capillary action reduces heat loss due to excessive wetting and balances the water supply. To optimize thermal energy utilization, a thermoelectric generator (TEG) module was integrated so that electricity could be generated from the temperature gradient spanning the TEG. A multifunctional system was developed to evaporate water and generate electricity concurrently. The hybrid system achieved an evaporation rate of 1.52 kg m^−2^ h^−1^ and a maximum power density of 66.73 mW m^−2^ under 1 kW m^−2^ solar light irradiation. The fabric produced using a weaving process for scalability demonstrated potential for large‐scale desalination and power generation in outdoor settings, which opens new possibilities for the development of efficient, scalable, and reliable solar water‐power cogenerators.

## 2 Results and Discussion

### 2.1 Design and Fabrication of CBFG

**Figure 1:**
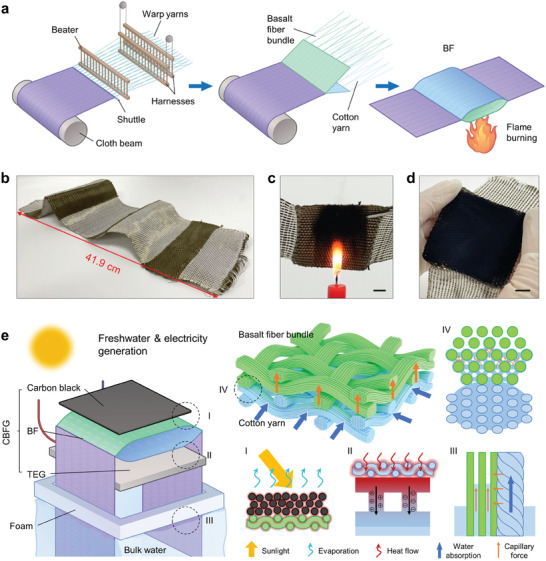
Design and fabrication of the CBFG. a) Preparation process of the CBF comprising weaving and carbon black deposition. b) Photograph of a 60‐cm‐long BF fabric. c) Deposition of carbon black on the BF surface by flame burning. Scale bar: 1 cm. d) A piece of 6 × 6 cm^2^ CBF. Scale bar: 1 cm. e) Structure and function for each part of the CBFG.

A bilayer hybrid fabric consisting of basalt fibers and cotton yarns (BF) was first prepared using an industrial loom. The fabrication of BF is shown in **Figures** **1**a and S1 (Supporting Information). BF consists of two consecutive fabric structures. The first structure comprises hydrophilic cotton yarns and basalt fiber bundles arranged in alternation along the warp direction and cotton yarns arranged as wefts. This structure is used for water transport. The second structure has a double‐layer arrangement in which the basalt fiber bundles and cotton yarns used as warp threads are divided into upper and lower layers by a programmed design in the loom. Fibers of the same material are used for the weft arrangement. The BF can be easily expanded with different warp thread numbers and weft thread widths (Figure 1b). The dimensions of BF depend on the size of the machine (Figure S2, Supporting Information). The BF basalt fabric was covered with a thin film of carbon black via flame cauterization (CBF) (Figure 1c,d). The prepared CBF exhibited good flexibility and foldability (Movie S1, Supporting Information).

The CBF‐based cogenerator (CBFG) system for efficient water evaporation and power generation in this study is shown in Figure 1e. CBF and the TEG are embedded in expandable polyethylene foam so that the entire system can float on the water surface. The generation, transport, and utilization of heat flow in the CBFG involves four parts: i) solar energy is collected and converted into heat by the carbon black layer, which has a high light absorption capacity; ii) waste heat from the bottom of the CBF flows through the TEG for power generation; iii) sufficient water supply is ensured through the excellent water absorption of the cotton yarn; and iv) the adsorption of a small amount of water in the cotton yarn by capillary action in the adjacent basalt fiber bundles reduces heat loss caused by excessive wetting. The energy input is provided by sunlight, whereas the carbon black film covering the top layer facilitates efficient light–heat conversion. The low thermal conductivity of the basalt fibers promotes the confinement of most of the solar heat to the CBF surface for water evaporation.^[^ ^34^, ^35^ ^]^ The low‐grade waste heat generated in the CBF region is subsequently transferred to the TEG and used for power generation through the Seebeck effect due to the thermal gradient with the water below.^[^ ^36^ ^]^ This ensures that solar light‐induced heat generation in the system is utilized to maximum.

### 2.2 Characterization of CBF

**Figure 2:**
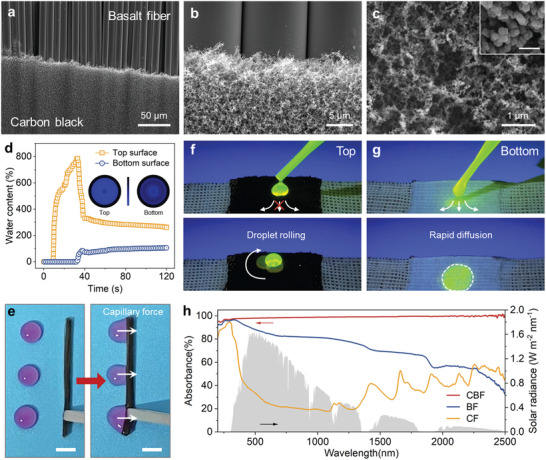
Characterization of CBF. a–c) Micromorphology of basalt fiber bundles after carbon black deposition. The inset is an enlarged SEM image of the carbon black. Scale bar: 100 nm. d) MMT test results for the relative water content at the upper and lower surfaces of BF with the cotton yarn layer facing up. The inset in the figure is the water content distribution on the fabric surface at 120 s. Blue and black indicate high and low contents, respectively. e) Capillary effect of basalt fiber bundles. Scale bar: 5 mm. f, g) Fluorescence image reflecting the asymmetric wettability of the top and bottom layers in CBF. Droplets (stained with 0.1 wt% sodium fluorescein) contacting the top and bottom layers of CBF under ultraviolet irradiation, respectively. h) Absorption spectra of fabric samples from 200 to 2500 nm.

The microscopic morphology of CBF was investigated using scanning electron microscopy (SEM). The upper and lower layers in the middle region of CBF are separate plain structures formed by basalt fiber bundles and cotton yarns, respectively (Figure S3, Supporting Information). The two end regions are mixed plain structures with interwoven fibers, which allows the fluid transport to each other (Figure S3b, Supporting Information). The low thermal conductivity of basalt and cotton yarn facilitates thermal localization in CBF. The top of BF remained at a low temperature even when its bottom was fixed to a heater (50 °C) (Figure S4, Supporting Information). The basalt fiber bundle consists of thousands of individual basalt fibers (diameter of ≈13 µm). Burning with a candle flame resulted in the formation of a carbon black layer with a thickness of ≈14 µm on the surface of the fibers, which consisted of loosely aggregated nanoparticles with an average diameter of ≈37 nm (**Figure** **2**a–c; Figure S5a, Supporting Information). The 3D optical microscopy image showed the roughness of the carbon black layer (Figure S5b, Supporting Information). The disordered nature of the carbon black particles was confirmed using Raman spectroscopy (Figure S6, Supporting Information). The porous structure of the carbon black layer ensures that water vapor can escape rapidly.

The moisture management test (MMT) was used to characterize the interlayer water transport properties of CBF (Figure S7, Supporting Information), which are critical for solar interface evaporation. The variation in the relative water content between the two sides of the CBF sample indicates that the water content at the top layer stabilized at 1200% when the carbon black layer was facing up (Figure S8, Supporting Information). The water content of the bottom layer was constantly zero. This indicates that water collected at the top layer. The nanorough structure of the carbon black layer makes the surface hydrophobic.^[^ ^37^ ^]^ When CBF was immersed in water, a bubble film was observed on the surface of the carbon layer because of its hydrophobicity (Figure S9, Supporting Information). Water transport between the layers was evaluated by turning BF so that the cotton yarn layer faced upward. As shown in Figure 2d, after 30 s of active water supply, the water content at the top layer increased to 785%, which is attributed to the excellent water‐absorbing properties of the cotton yarn. Subsequently, the water content at the top layer decreased rapidly to 354%, whereas that at the bottom layer increased to 82%. Notably, the water content at the upper and lower surfaces changed almost simultaneously. Because of the core absorption effect, some of the water adsorbed by the cotton yarns was rapidly transferred to the basalt fiber bundles. The significantly smaller water content at the lower layer compared to that at the upper layer indicates that the fabric structure of the two‐layer blend prevented excessive wetting. The water content of the top layer can be further regulated by adjusting the weaving density of the cotton yarn layer (Figure S10 and Table S1, Supporting Information). Figure 2e shows the wetting process of the basalt fiber bundles. When a water droplet was added to a basalt fiber bundle, it rapidly diffused (within seconds) into the bundle owing to capillary force. The gaps between the individual fibers in the bundle (width: 10–60 µm, Figure S11, Supporting Information) are crucial for achieving the desired wettability.^[^ ^38^ ^]^ Fluorescent water droplets were used to visualize the asymmetric CBF water transport. The fluorescent water droplets diffused rapidly in the cotton yarn layer but did not continue to diffuse into the bottom layer when the carbon black layer faced upward (Figure 2f,g). The significant hydrophobicity of the carbon black layer caused the droplets to roll easily on its surface (Movie S2, Supporting Information). We also investigated the light absorption properties of different fabric samples. As shown in Figure 2h, the measured spectrum of CBF indicates relatively high absorption in the visible region, which exceeds 98.1%; the absorption is 98.7% in the short‐wave near infrared (NIR) region and 99.5% in the long‐wave NIR region. The average absorption efficiency of CBF from the visible region to the NIR region is 99.1%. In comparison, cotton fabric (CF) and BF exhibited low absorption efficiencies (34.3% and 70.2%, respectively). CBF is therefore suitable as a photothermal material.

### 2.3 Photothermal Evaporation Performance of CBFG

**Figure 3:**
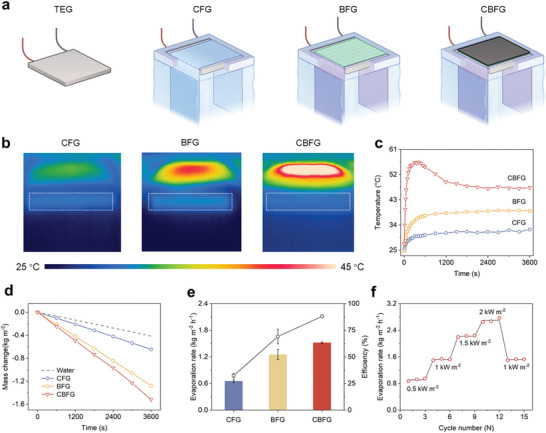
Photothermal conversion and evaporation performance of fabric evaporators. a) Schematics of the water and power cogeneration systems, including TEG, CFG, BFG, and CBFG. b) Infrared thermal images of the three systems at steady state. c) Average surface temperatures of the three systems. d) Mass change of the systems within 1 hour of 1 kW m^−2^ irradiation. e) Evaporation rates and energy conversion efficiencies of the three systems. f) Evaporation rates of CBFG under different solar power density cycles.

To achieve continuous steam–electricity cogeneration, a system combining the photothermal evaporation of water with waste heat utilization was designed. Three systems based on different fabrics and a TEG were prepared (**Figure** **3**a) comprising a i) CF‐based cogenerator (CFG), ii) BF‐based cogenerator (BFG), and iii) CBFG. A customized measurement system was built to evaluate the water and electricity cogeneration performance (Figure S12, Supporting Information). The surface and side temperatures of the samples were measured using infrared thermal image. The samples exhibited a temperature gradient from the surface to the bottom at steady state (Figure 3b). Three systems demonstrate excellent ability to localize heat at the surface. In addition, the TEG regions of all three systems were significantly hot because the utilization of residual heat from the upper fabric layer by the TEG prevented effective heat conduction to the bulk water. The variations in the average surface temperatures of CBFG, CFG, and BFG after irradiation were recorded (Figure 3c). Consistent with the spectral absorption results, the surface temperature of CBFG was significantly improved and the photothermal conversion performance better compared to that of CFG and BFG. It is noteworthy that the surface temperature of CBFG exhibited a rapid increase to 56.4 °C, which is attributed to the outstanding photothermal effect of the carbon black layer. The surface temperature then decreased gradually owing to the wetting of the basalt fiber layer until it reached a steady‐state temperature of ≈47.3 °C.

The solar evaporation performance of the systems was evaluated by recording the change in water mass over the solar irradiation time. As shown in Figure 3d, the water mass decreased linearly with the solar irradiation time. The evaporation rate of CBFG (1.52 kg m^−2^ h^−1^) is 21.6% higher than that of BFG (1.25 kg m^−2^ h^−1^) and 3.6 times that of pure water (0.42 kg m^−2^ h^−1^). The water evaporation efficiency (*η*) was calculated using Equation S2 in Note S2 (Supporting Information). As expected, CBFG exhibited a higher water evaporation efficiency (88.06%) than BFG (69.15%) and CFG (32.35%) (Figure 3e). The cycling performance of CBFG was evaluated by measuring its evaporation rate under cyclic solar irradiation. CBFG maintained a stable evaporation rate after 15 cycles under irradiation at different light intensities (Figure 3f). In addition, its evaporation rate was essentially stable over 15 days of 1 kW m^−2^ solar irradiation for 1 h per day (Figure S13, Supporting Information). After 15 days of evaporation, no significant changes were observed in the carbon black layer on the surface of CBFG in the wetted state (Figure S14, Supporting Information). These results indicate that CBFG has excellent cyclic stability over repeated use. In addition to pure water, the evaporation performance of saline solutions was investigated to evaluate the salt endurance and salt‐rejecting capability of the CBFG. As shown in Figure S15 (Supporting Information), the evaporation rate of NaCl solutions is slightly lower than that of pure water. The evaporation rate decreases with increasing salinity, which is due to the lower saturated vapor pressure at higher salt concentrations.^[^ ^39^ ^]^

### 2.4 Power Generation Performance of CBFG

**Figure 4:**
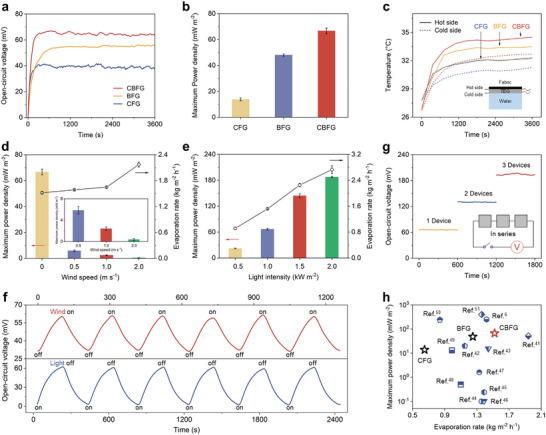
Power generation performance of the CFG, BFG and CBFG systems. a) Open‐circuit voltage–time curves of the three systems. b) Maximum power density of the three systems. c) Temperature‐time curves of the hot and cold sides of the TEG in the three systems. d, e) Water–electricity cogeneration performance of CBFG under different wind speeds and light intensities. f) Open‐circuit voltage curves under wind and light on/off cycles. g) Open‐circuit voltage curves of three CBFG systems connected in series. h) Comparison of our work with other water–electricity cogeneration systems.

CFG and BFG were used as controls to evaluate the power generation ability of CBFG. CBFG achieved an open‐circuit voltage of 65.7 mV and short‐circuit current of 3.6 mA, which are higher than those of CFG (39.8 mV and 1.3 mA) and BFG (54.9 mV and 3.2 mA) (**Figure** **4**a; Figure S16, Supporting Information). Moreover, the stability of CBFG was demonstrated by the absence of significant changes in voltage or current under continuous light irradiation. The calculated maximum power density of CBFG under 1 kW m^−2^ illumination is 66.7 mW m^−2^, which is 383.3% and 38.7% higher than that of CFG (13.8 mW m^−2^) and BFG (48.1 mW m^−2^), respectively (Figure 4b). The improved power density and evaporation rate can be attributed to the excellent light‐absorbing and heat‐generating properties of the carbon black layer and thermal localization due to the basalt fabric layer. Figure 4c and Figure S17 (Supporting Information), respectively, show the temperature variation and difference at the hot and cold ends of the TEG in the different samples. The residual temperature transferred to the TEG through CBF thermal insulation is 34.3 °C. The temperature difference between the hot and cold sides of the CBFG system (1.9 °C) is higher than that of the CFG (1.1 °C) and BFG (1.3 °C) systems. The ability of the TEG to harvest electrical energy through the Seebeck effect due to the low‐grade residual heat generated at the bottom of the fabric and the temperature difference relative to bulk water maximizes energy utilization in the system.^[^ ^36^ ^]^

Because the natural environment is always changing, we analyzed power generation in CBFG under different irradiation intensity and wind speed conditions. We first explored the performance of the CBFG electricity–water cogeneration system under different wind speeds. As the external wind speed increased from 0 to 2 m s^−1^, the evaporation rate of CBFG increased from 1.52 to 2.17 kg m^−2^ h^−1^ (Figure 4d; Figure S18a, Supporting Information). However, the open‐circuit voltage decreased from 64.6 to 8.1 mV (Figure S18b, Supporting Information), the short‐circuit current decreased from 3.6 to 0.2 mA (Figure S18c, Supporting Information), and the maximum power density decreased sharply to 0.4 mW m^−2^ (Figure 4d). The wind speed had a significant effect on the open‐circuit voltage. The relationship between the wind speed and output power was further verified in wind on/off cyclic tests. When wind was present, the voltage and current decreased rapidly. When the wind stopped, the voltage and current increased rapidly (Figure 4f; Figure S19a, Supporting Information). At larger wind speeds, thermal convection between the CBFG surface and the air is accelerated, leading to increased heat loss.^[^ ^40^ ^]^ The reduction in surface temperature results in decreased heat transfer to the TEG, which decreases the temperature difference between the two sides of the TEG, ultimately causing a significant decline in power generation (Figure S20, Supporting Information). These results indicate that CBFG power generation is suitable under light or no wind conditions. We also investigated the water–power generation performance of the CBFG system at light intensities of 0.5–2 kW m^−2^. As the light intensity increased from 0.5 to 2 kW m^−2^, the open‐circuit voltage of CBFG increased from 42.1 to 103.4 mV and the evaporation rate increased from 0.92 to 2.73 kg m^−2^ h^−1^ (Figure 4e; Figure S21, Supporting Information). The calculated maximum power density of 187.4 mW m^−2^ was obtained under 2 kW m^−2^ solar irradiation. The evaporation rate and maximum power density varied linearly with the light intensity. CBFG showed good reproducibility in the light cyclic experiments (Figure 4f; Figure S19b, Supporting Information). Multiple CBFGs can be connected in series to increase the voltage and power produced. As shown in Figure 4g, the voltage increased to 193.5 mV when three CBFG systems were connected in series. We summarized and compared the performance of CBFG, BFG, and CFG with that of previously reported water‐power cogeneration systems (Figure 4h; Table S2, Supporting Information).^[^ ^6^, ^41^, ^42^, ^43^, ^44^, ^45^, ^46^, ^47^, ^48^, ^49^, ^50^, ^51^ ^]^ The comparison results show that, under 1 kW m^−2^ solar irradiation, the integrated cogeneration performance of the CBFG is better than that of some previous reports.

### 2.5 Large‐Scale CBFG for Practical Applications

**Figure 5:**
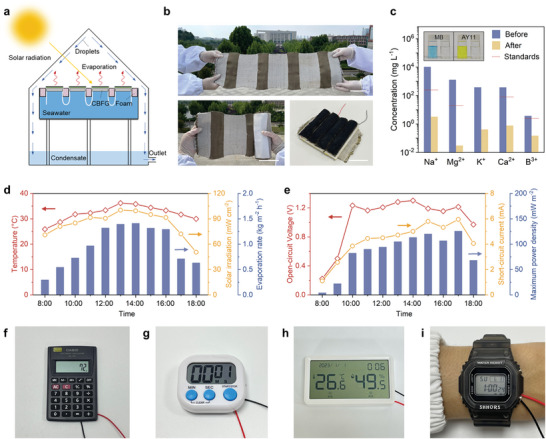
Desalination and power utilization using large‐scale CBFG. a) Experimental setup for desalination and its operating mechanism. b) Photograph of the large‐scale CBFG system. Scale bar: 10 cm. c) Ion concentration of seawater before and after purification. The insets are photographs of the dye solution before and after purification. d) Variations of evaporation rates and ambient conditions during daytime. e) Variation of power generation performance of the outdoor system. f–i) Photographs of the system powering a calculator, a timer, a thermometer, and a watch, respectively.

We also demonstrated the feasibility of using CBFG for water and power cogeneration applications in outdoor environments. The scalable manufacture of CBFG can be flexibility achieved using advanced textile technology.^[^ ^52^ ^]^ Large‐scale CBFG for practical applications was easily prepared by expanding the width of the warp and weft yarns using a loom (Figure 5b).

**Figures** **5**a and S22 (Supporting Information) show the experimental setup for the outdoor CBFG system. Solar radiation was absorbed by CBFG floating on the surface of water to generate water vapor. The vapor was condensed on the top surface and collected in a tank as clean water, which can be used for drinking, agriculture, and other purposes. Simultaneously, evaporation waste heat was converted into electricity as the output from the TEGs in series (Figure S23, Supporting Information). The device was placed in an open space at Wuhan Textile University and operated for 11 h from 7:30 to 18:30 for evaporation and power generation using seawater from the Yellow Sea. As shown in Figure 5d,e, the ambient temperature and solar intensity exhibited consistent trends. The evaporation quality and electrical output signal varied with the weather conditions. The system produced ≈10.6 L m^−2^ of freshwater per day and generated ≈0.9 Wh m^−2^ of electricity. Owing to the physical and chemical stability of the textile, the CBFG‐based evaporation and purification device can be operated with various liquids, including saline water, dyeing wastewater, and heavy metal solutions. CBFG exhibited a strong ability to remove dyes and heavy metal ions from wastewater and seawater (above 99.9%) (Figure 5c; Figure S24, Supporting Information). Moreover, during prolonged purification processes, the surface of the CBFG does not exhibit crystallization or solid formation, which contributes to its stable operation (Figure S25, Supporting Information). The electricity generated by the system could drive a calculator, timer, thermometer, or watch (Figure 5f–i; Movie S3, Supporting Information). These results demonstrate the potential practical applications of CBFGs in solar‐driven evaporation and thermal power generation.

## 3 Conclusion

We fabricated CBFG for cogenerating water vapor and thermoelectricity from basalt fiber and cotton yarn using a textile weaving technique. The fabric has a bilayer structure in which the cotton yarn in the lower layer ensures adequate water supply while the basalt fibers in the upper layer facilitate efficient photothermal conversion, thermal localization, and balanced water supply after carbon deposition. An evaporation rate of up to 1.52 kg m^−2^ h^−1^ was achieved. When combined with a thermoelectric module, the hybrid system generated a maximum output power density of 66.73 mW m^−2^. In outdoor experiments, the 400 cm^2^ scalable fabric achieved a freshwater yield of 10.6 L m^−2^ and power production of 0.9 Wh m^−2^. The electricity produced could power electronic devices such as electronic calculators, timers, thermometers, and watches. The use of traditional textile technology makes fabric‐based water and electricity cogenerators feasible for large‐scale production. The system has considerable potential in various applications such as solar desalination, wastewater treatment, and power generation.

## 4 Experimental Section

### Materials

Basalt fiber bundles were purchased from Haining ANJIE Composite Materials Co., Ltd. TEGs (TES1‐12701 SR) were purchased from Aladdin. Cotton yarn, reactive red 195 (RR 195), acid yellow 11 (AY 11), and methylene blue (MB) were provided by Luthai Textile Co., Ltd. Sodium fluorescein, NiCl_2_‐6H_2_O, CuCl_2_‐2H_2_O, CdCl_2_‐2H_2_O, BaCl_2_‐2H_2_O, and other chemicals were provided by Sinopharm Chemical Reagent Co., Ltd. Candles were purchased from the local supermarket. Seawater was collected from the Yellow Sea. Deionized water was used for all experiments unless otherwise stated.

### Preparation of the CBF

Basalt fiber bundles and cotton yarns were weaved into fabrics using an SGA 598 semiautomatic loom (manufactured by Ningbo Textile Instrument Factory). During the weaving process, basalt fiber bundles and cotton yarns were alternately passed through loom heddle frames and reeds of the loom as tension warp yarns. The ends of the fabric were of a single‐layer construction; cotton yarns as weft yarns were inserted into the tension warp. The middle portion of the fabric was a two‐layer structure, woven using basalt fiber bundles and cotton in the upper and lower layers, respectively. The number of warp and weft yarns was adjusted according to the desired size of the fabric. BF was then molded. The BF was placed above the candle flame (at ≈2.5 cm from the flame core) at a surface temperature of 320–450 °C. The position was continuously adjusted until the basalt fiber layer completely adhered to the carbon black, and the CBF was obtained. The specific fabric structure and weaving flowchart are shown in Figure S1 (Supporting Information). The cotton yarns were used as warp and weft yarns and interwoven to obtain a single layer of plain weave CF as the control group.

### Solar Evaporation Measurement

The evaporation was monitored in real‐time using a CBFG as a solar evaporation device placed on an electronic balance (ME204E, Mettler Toledo, Switzerland) and illuminated by a solar simulator (CELHXF300, Education Au‐light Co Beijing, China). The light intensity magnitude was corrected by an optical power meter (CEL‐NP2000‐2A, Education Au‐light Co Beijing, China). Temperatures on the fabric surface and sides were monitored using an infrared thermometer (E8‐XT, FLIR, USA).

### Power Generation Measurement

The TEG was fixed in the foam with the hot end touching the bottom surface of the fabric and the cold end touching the water to generate electricity using the temperature difference between the two ends. The real‐time open‐circuit voltage and short‐circuit current of the TEG were recorded with a multimeter (6500, Keithley, USA). Temperature variations on the upper and lower surfaces of the TEG were monitored with a thermocouple digital temperature probe (TA612C, Suzhou TASI Electronics Co., Ltd., China). Airflow was generated by an electric fan, and airflow velocity was measured by an anemometer (PM6252B, Guilin Huayi PeakMeter Technology Co., Ltd., China).

### Characterization

The morphologies of the samples were analyzed by SEM (GeminiSEM 300, ZEISS, Germany). The 3D optical microscopy image was acquired using a 3D laser microimaging system (VK‐X7000, Keyence, Japan). Raman spectra were acquired using a confocal Raman imaging microscope (LabRAM Odyssey, HORIBA, Japan). Absorptsion spectra of the fabrics were acquired using a spectrometer with an integrating sphere (UV‐2550, Shimadzu, Japan). The directional water transport properties were quantitatively measured with a MMT (SDL ATLAS, Ltd., USA). As the measurements were taken, the fabric was placed between the sensors on the top and bottom sides, and a drop of brine (0.9% NaCl) was applied to the upper surface of the fabric within 30 s. The concentration of metal ions was determined using inductively coupled plasma emission spectrometry (Agilent 5110, USA). Tests were performed at a relative humidity of ≈40% at 25 °C, unless otherwise stated.

## Conflict of Interest

The authors declare no conflict of interest.

## Supporting information



Supporting Information

Supplemental Movie 1

Supplemental Movie 2

Supplemental Movie 3

## Data Availability

The data that support the findings of this study are available from the corresponding author upon reasonable request.
