# Supplementary material for: Scalable Asymmetric Fabric Evaporator for Solar Desalination and Thermoelectricity Generation
Source: Adv Sci (Weinh). 2024 Sep 20;11(45):2406474. doi: 10.1002/advs.202406474 (PMC11615747; doi:10.1002/advs.202406474)
Supplement: Supplementary file 1 — Supporting Information [file ADVS-11-2406474-s004.pdf]

ADVANCED SCIENCE
Open Access

Supporting Information

for *Adv. Sci.*, DOI 10.1002/advs.202406474

Scalable Asymmetric Fabric Evaporator for Solar Desalination and Thermoelectricity Generation

Zhuan Fu, Dandan Zhong, Sijie Zhou, Leyan Zhang, Weihao Long, Jiajing Zhang, Xinyu Wang, Jiahao Xu, Jieyao Qin, Junyao Gong, Li Li, Liangjun Xia, Bin Yu* and Weilin Xu**

Supporting Information

Scalable asymmetric fabric evaporator for solar desalination and thermoelectricity generation

Zhuan Fu, Dandan Zhong, Sijie Zhou, Leyan Zhang, Weihao Long, Jiajing Zhang, Xinyu Wang, Jiahao Xu, Jieyao Qin, Junyao Gong, Li Li, Liangjun Xia, Bin Yu,* and Weilin Xu**

This PDF files includes:

Supplementary Materials

Movie S1

Carbon black deposition by flame burning: the carbon black was deposited on the surface of the CBF fabric by burning with a candle, after which the fabric maintained good flexibility.

Movie S2

Wettability of CBF: the water droplets exhibited asymmetric wettability in the top and bottom layers of the CBF.

Movie S3

Large-scale CBFG system powered the electronic devices: the CBFG system can power a calculator, a timer, a watch or a thermometer under simulated irradiation.

Figure S1 to S19

Note S1 and S2

Table S1

Legends for Movie S1 to S3

References

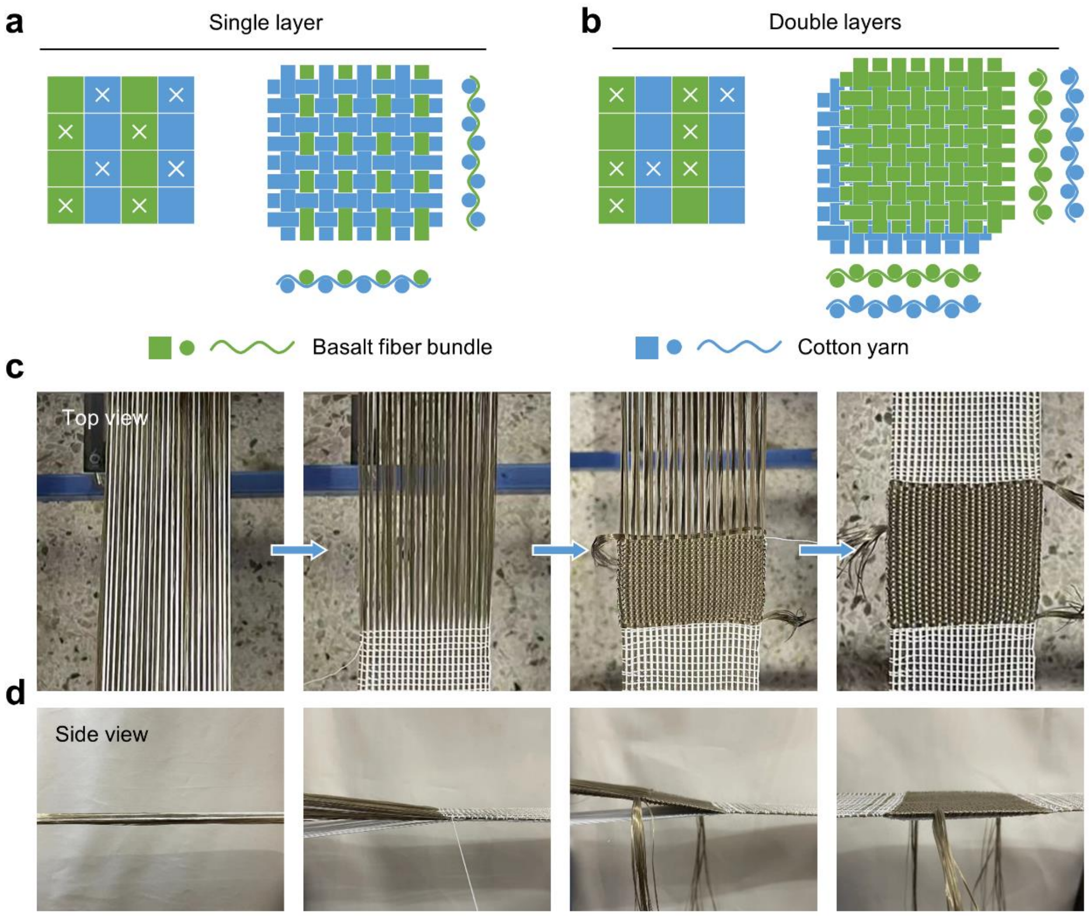

Figure S1. Weaving process of BF. a, b) Schematic diagram of lifting heddle and structure of BF. c, d) Photographs of the top and side during the weaving process.

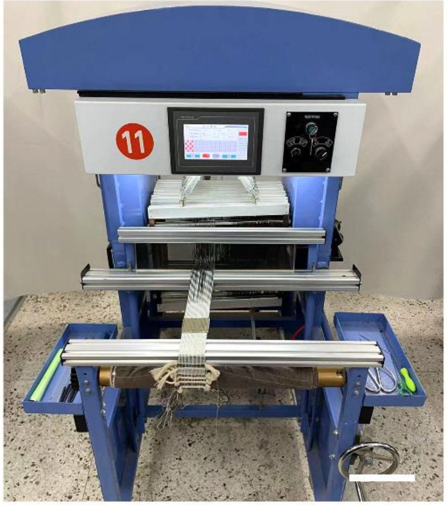

Figure S2. Photograph of the weaving loom used. Scale bar, 15 cm.

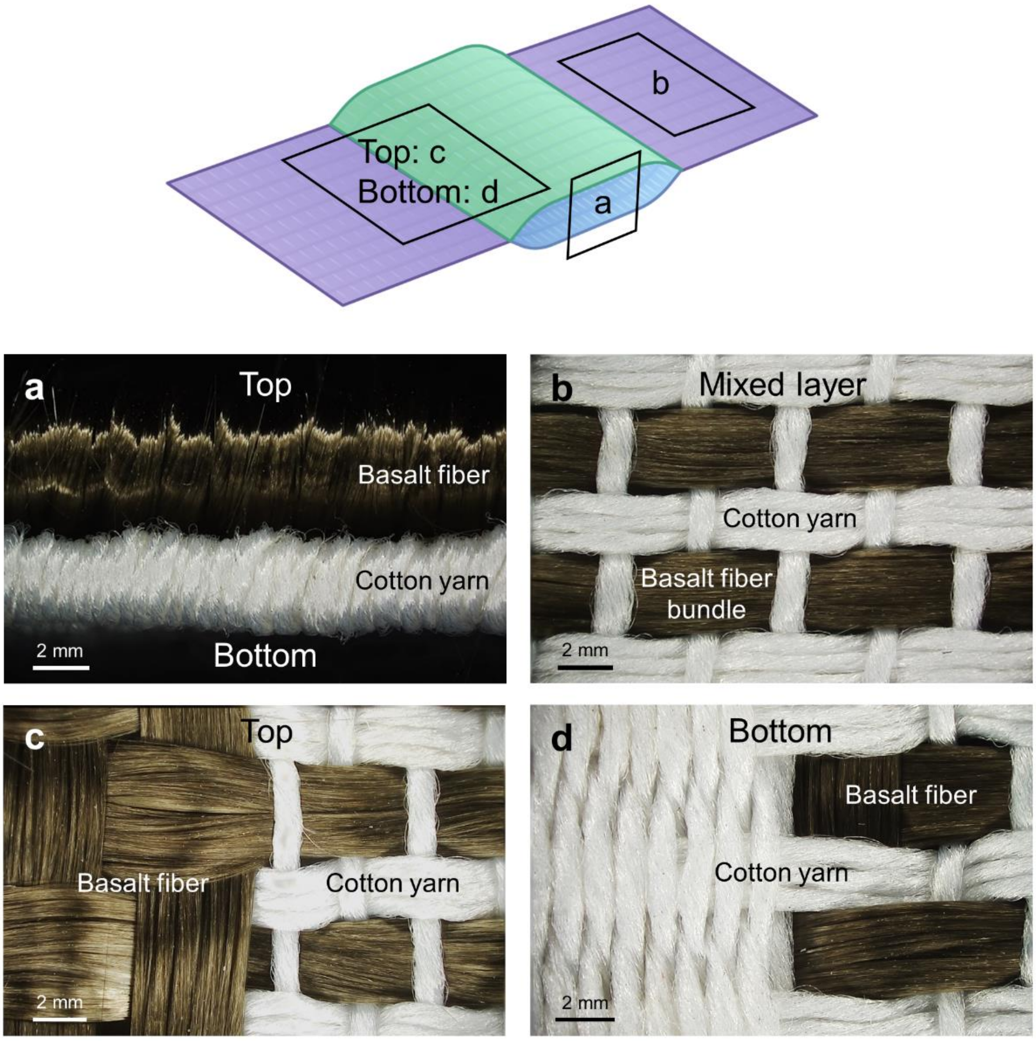

Figure S3. The optical micrographs showed the microstructure of BF. a) Bilayer structure of the BF. b) The structure of mixed layer with basalt fiber bundles and cotton yarns. c, d) Top and bottom structure at the interface of single and double layers.

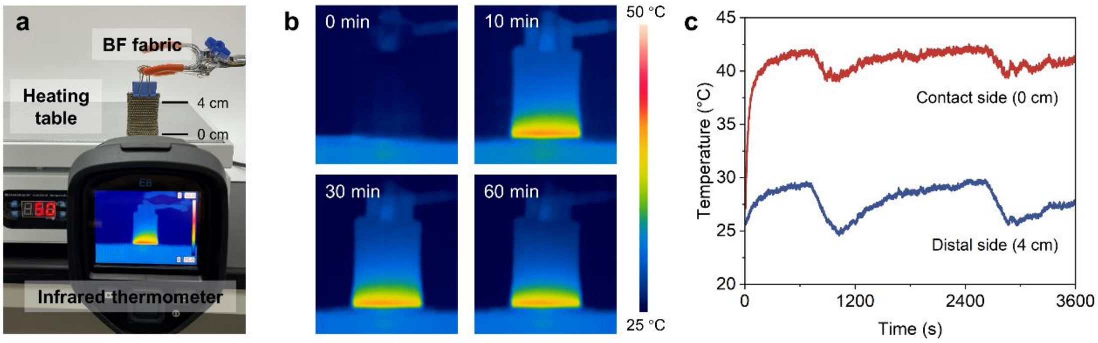

Figure S4. Thermal conductivity of the BF. a) Photograph of test equipment. b) Infrared images of the contact and distal sides of the BF. c) Corresponding temperature-time curves.

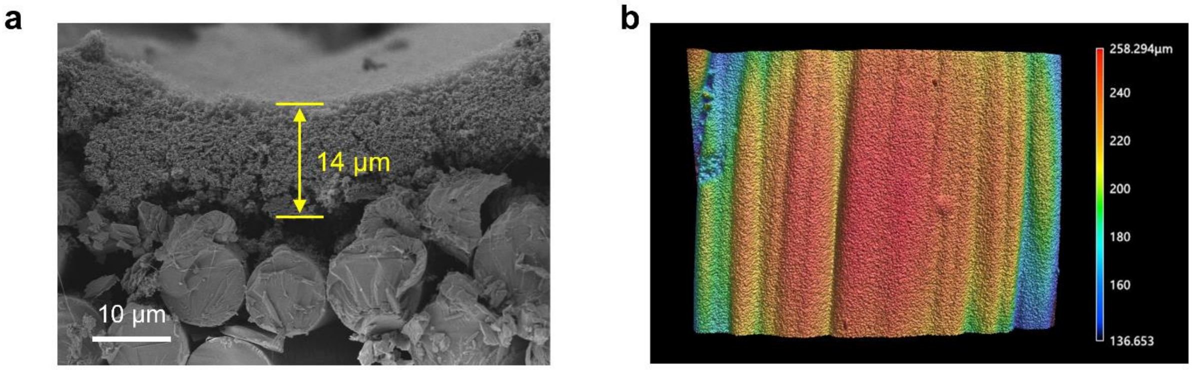

Figure S5. a) Cross-section SEM image of the carbon black deposited basalt fiber bundles. b) 3D optical microscope image of the carbon black deposited basalt fiber bundles.

Sdr value was used to analysis the roughness of the carbon black layer, which represents the ratio of the additional area generated by surface texture to the area defined by a plane. For example, a perfectly flat surface would have an Sdr value of 0. The results indicate that the carbon black layer exhibits a densely rough morphology, with an Sdr value of approximately 8.98.

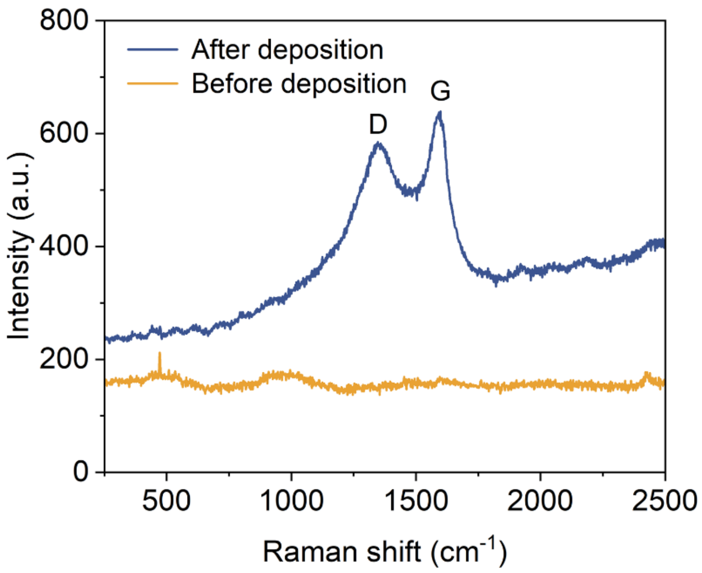

Figure S6. Raman spectra of basalt fiber layer before and after carbon black deposition.

After the deposition of carbon black, two distinct peaks were observed at 1598 cm^{-1} (peak G) and 1347 cm^{-1} (peak D), respectively. The G peak represents the in-plane bond stretching motion of C sp^2-type bond pairs. The D peak shows the presence of disorder in the carbon black. The results demonstrate that carbon black has a graphite-like structure with abundant disorder.[1]

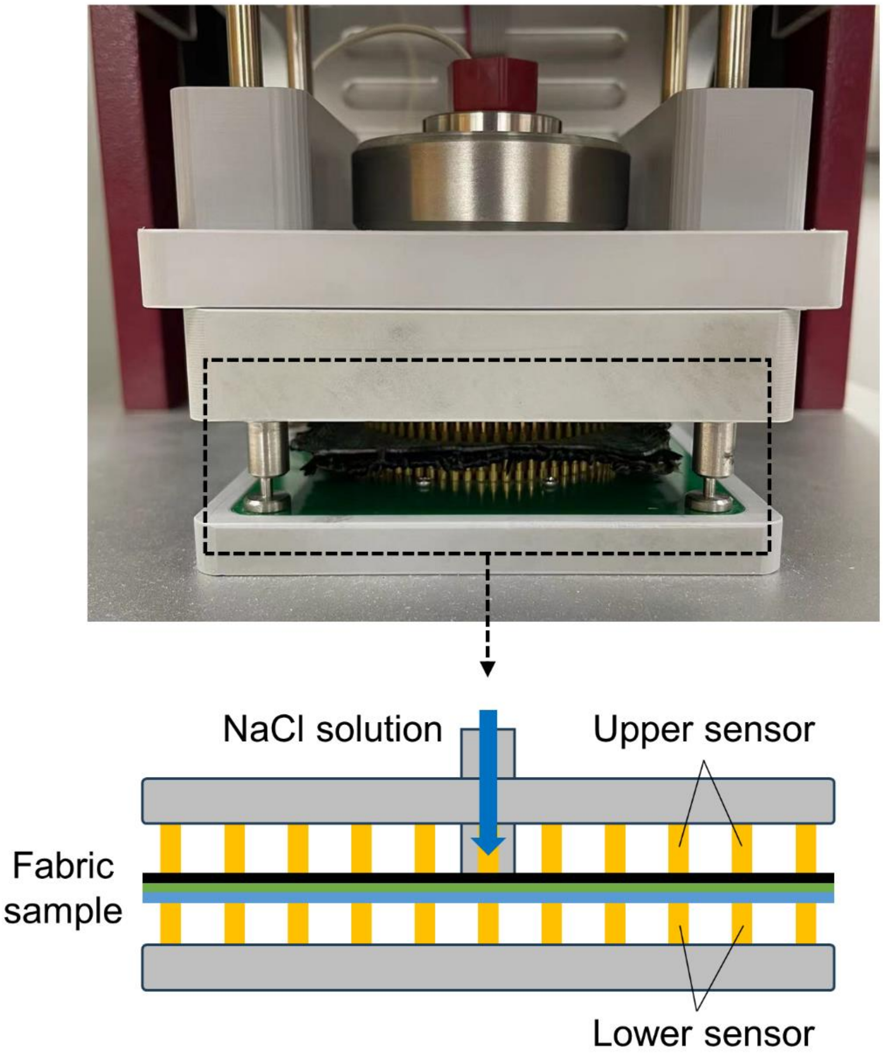

Figure S7. Photograph and structure schematic of the moisture management tester (MMT).

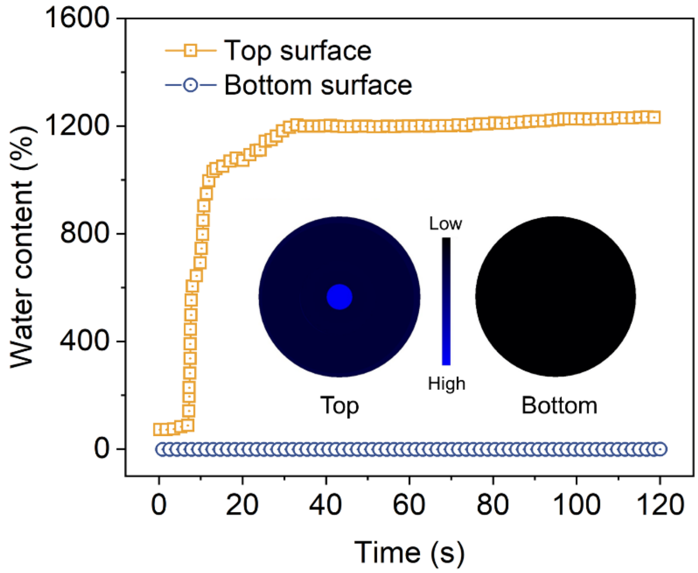

Figure S8. MMT measurement of the CBF with the carbon black layer facing up. The inset in the figure is the water content distribution on the fabric surface at 120 s.

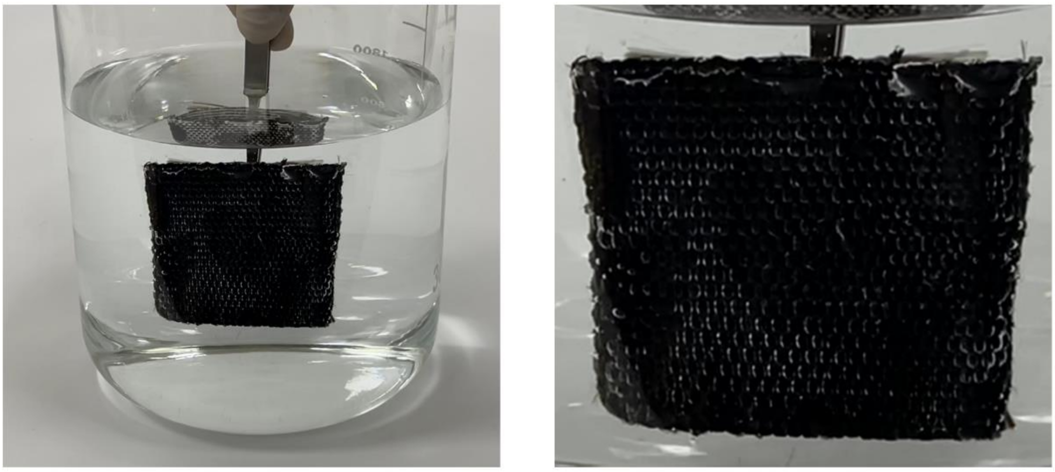

Figure S9. Optical photograph of the CBF immersed in water. The surface of the carbon black showed a distinct air layer.

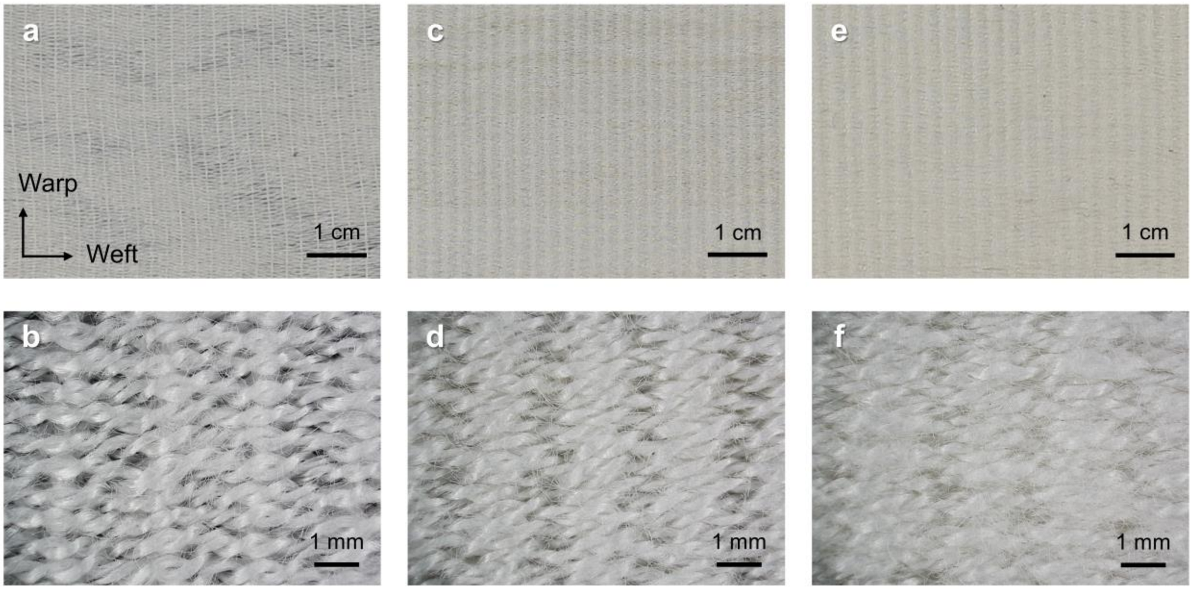

Figure S10. Optical photographs of cotton yarn layer with different densities, including a, b) 36×260, c, d) 108×500, and e, f) 180×800. (warp threads×weft threads)

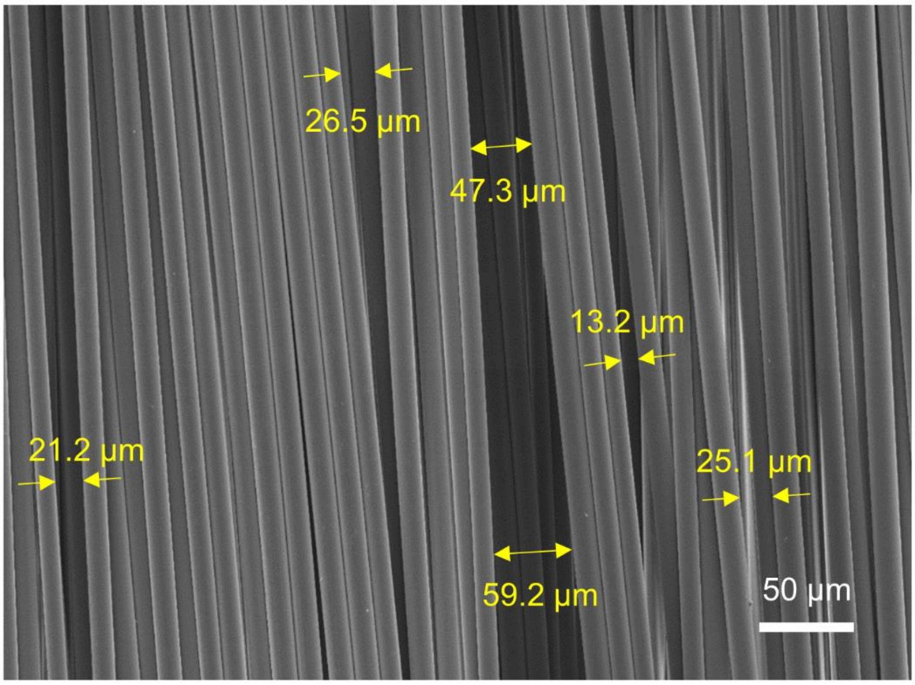

Figure S11. Gaps between individual fibers in basalt fiber bundles.

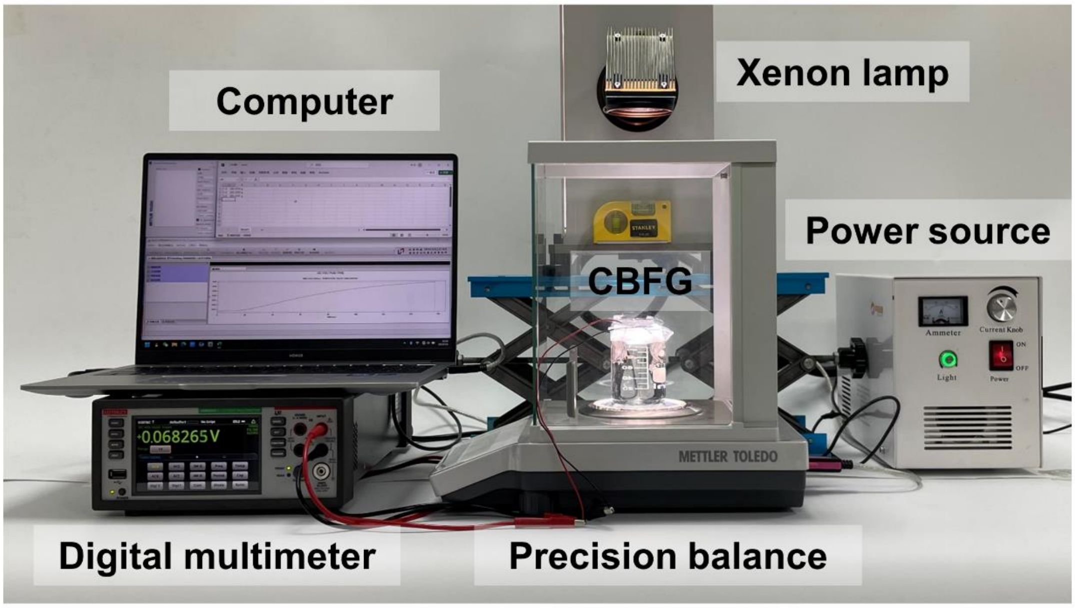

Figure S12. Photograph of the testing setup for evaporation and power generation.

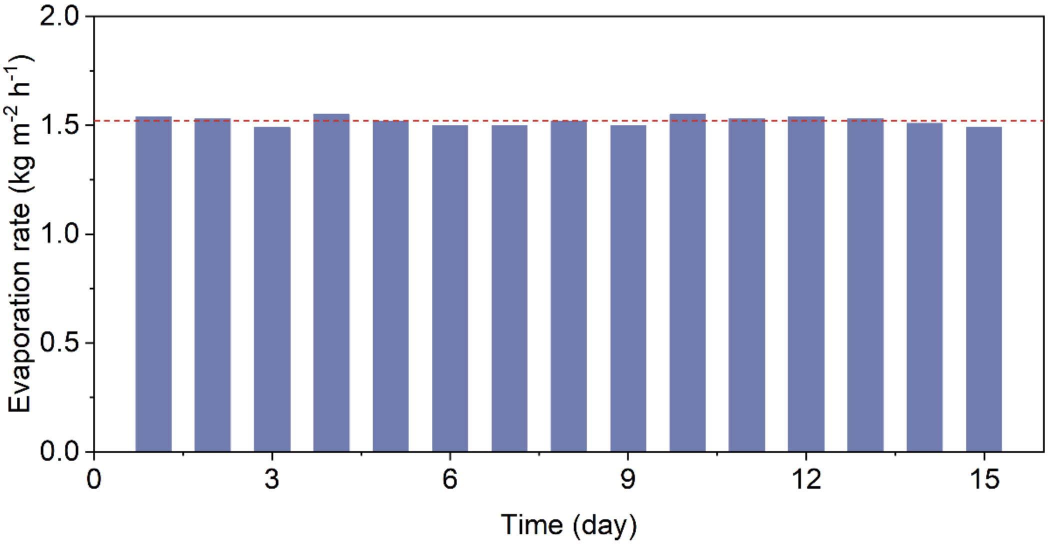

Figure S13. The long-time stability of the CBFG in water evaporation up to 15 days under 1 kW m^{-2} solar light irradiation for 1 h per day.

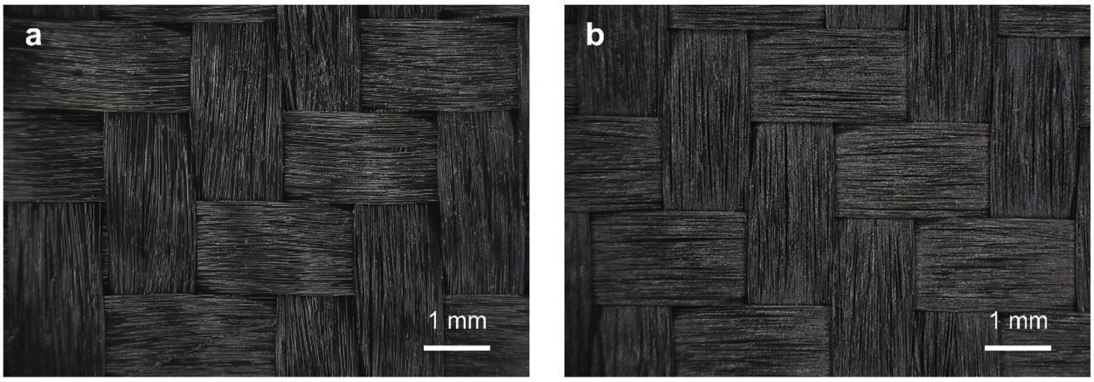

Figure 14. The 3D optical images of the top layer of the fabric in the wet state: a) before, and b) after 15 days of evaporation.

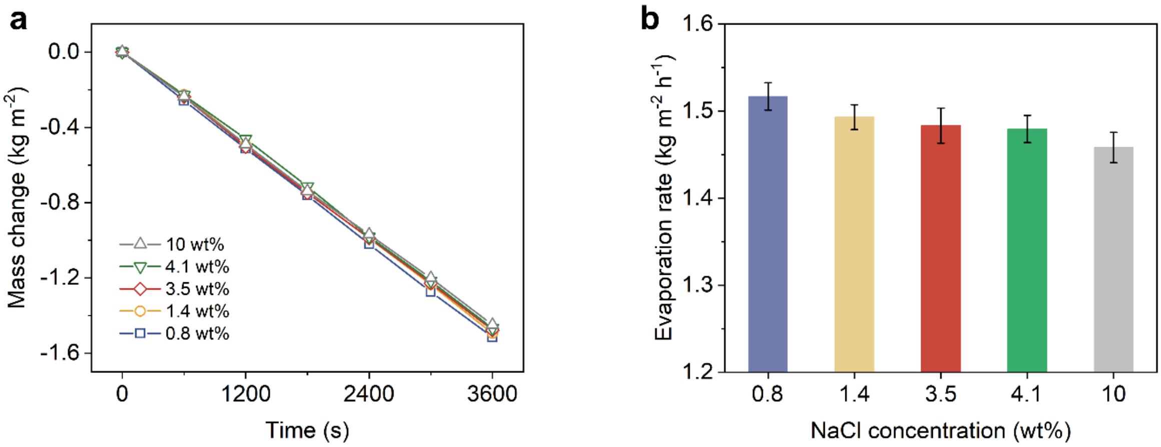

Figure S15. Evaporation performance of NaCl solution. a) Mass change and b) evaporation rate of different NaCl solutions.

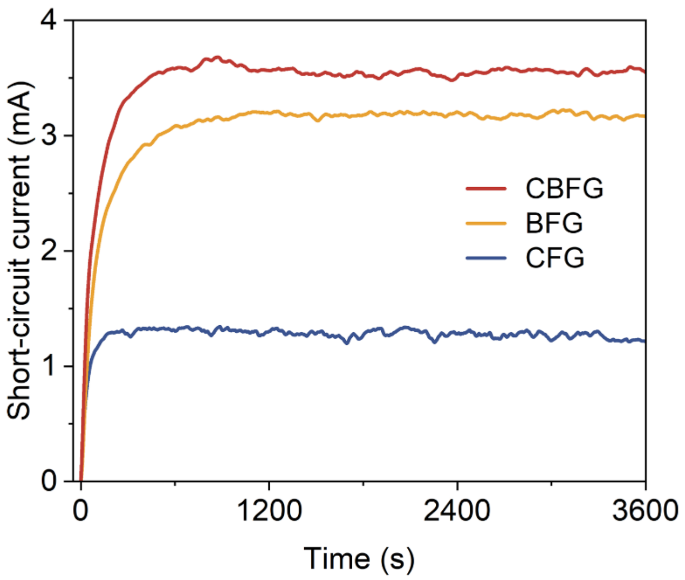

Figure S16. Short-circuit current-time curves of the three systems.

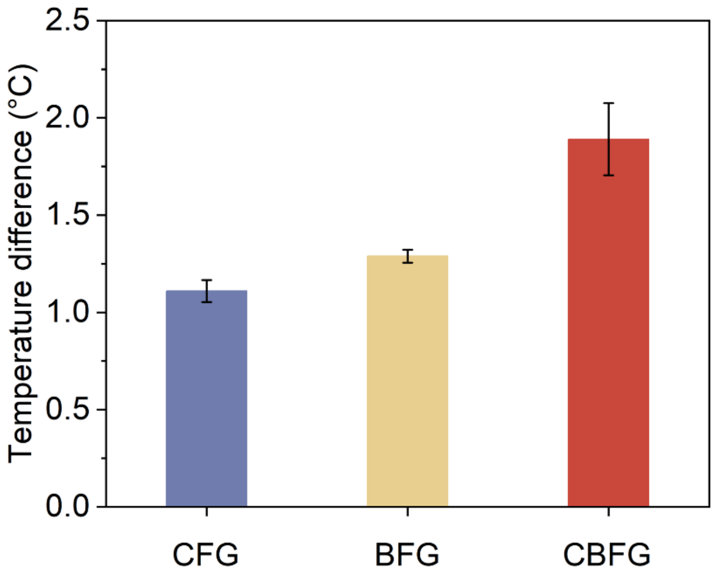

Figure S17. Temperature difference between the hot and cold sides of the TEG in the three systems.

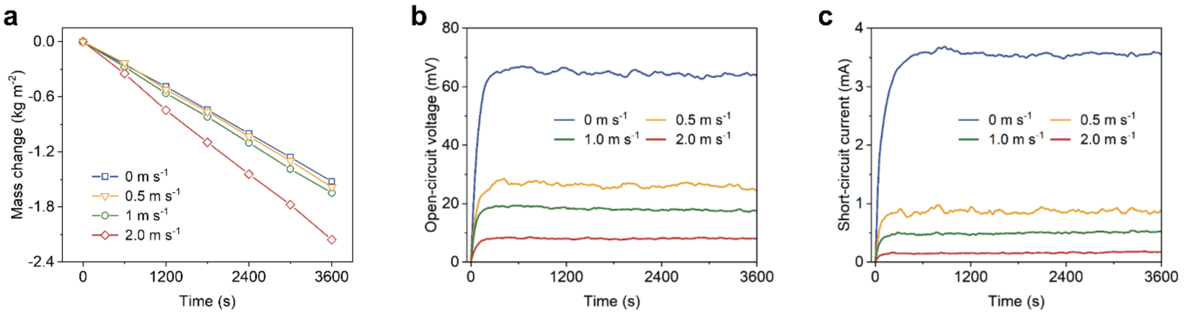

Figure S18. Variation of a) mass, b) open-circuit voltage, and c) short-circuit current with time for CBFG at different wind speeds.

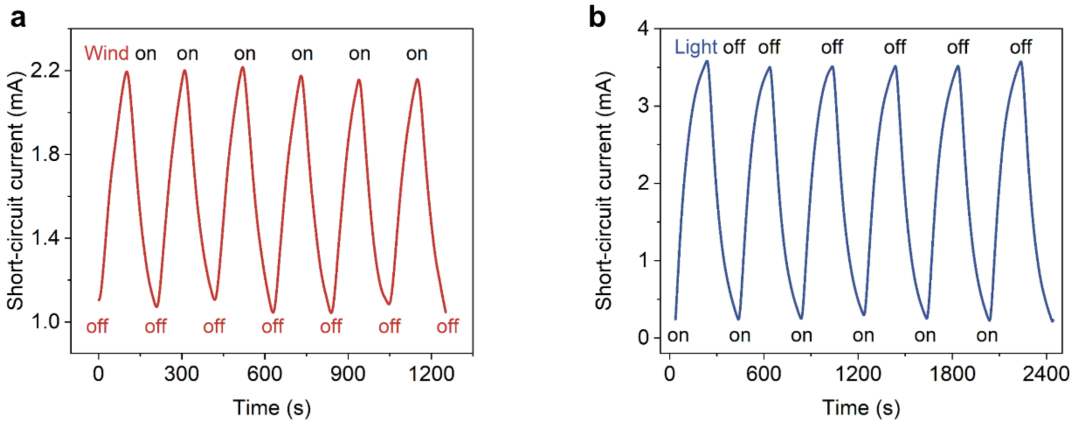

Figure S19. Short-circuit current curves of a) wind and b) light on-off cycles.

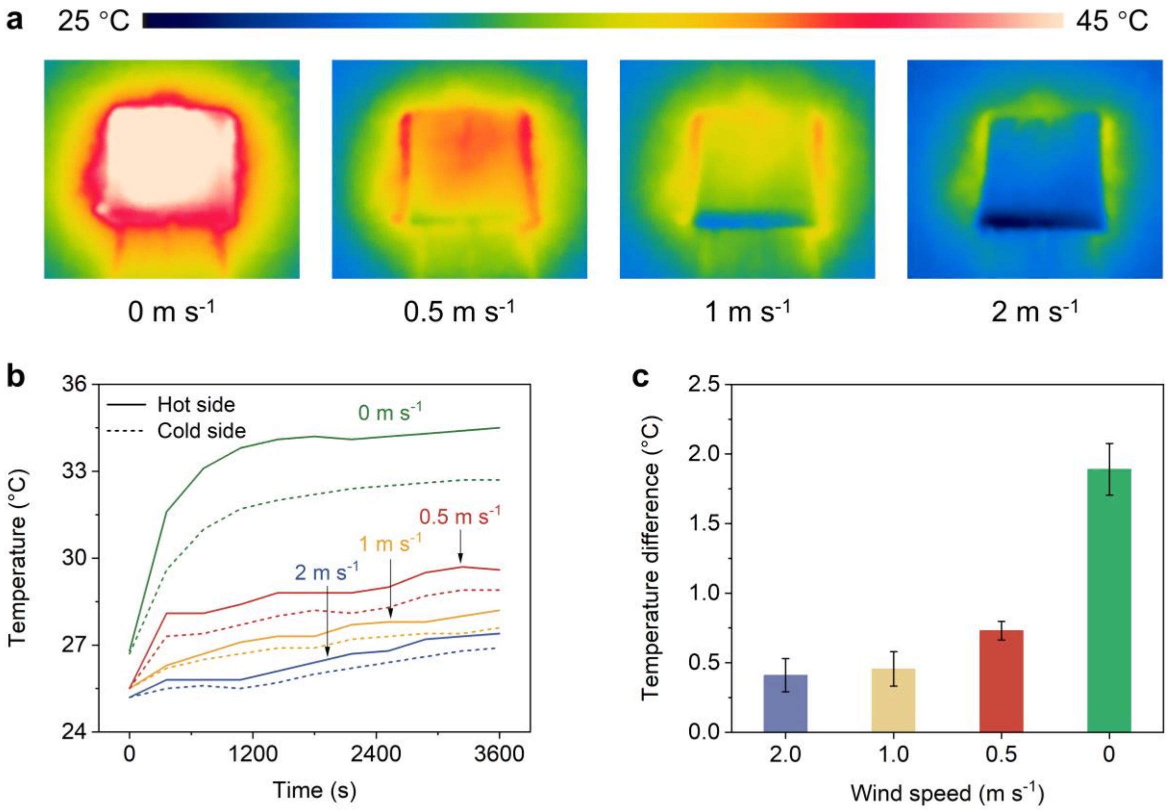

Appendix Figure 20. a) Infrared thermal images of the CBFG surface under different wind speeds. b) Temperature-time curves and c) Temperature difference at the hot and cold sides of the TEG under different wind speeds.

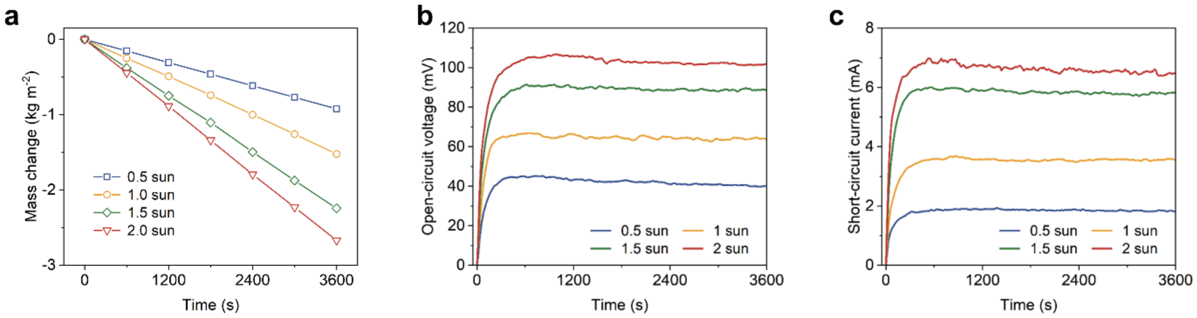

Figure S21. Variation of a) mass, b) open-circuit voltage, and c) short-circuit current with time for CBFG at different light intensities.

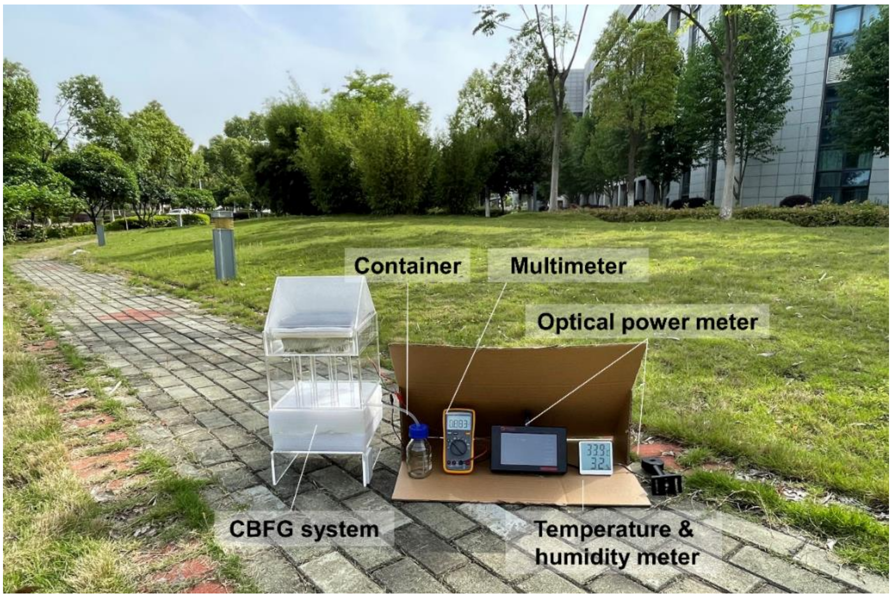

Figure S22. Outdoor equipment for solar water and electricity cogeneration and data recording.

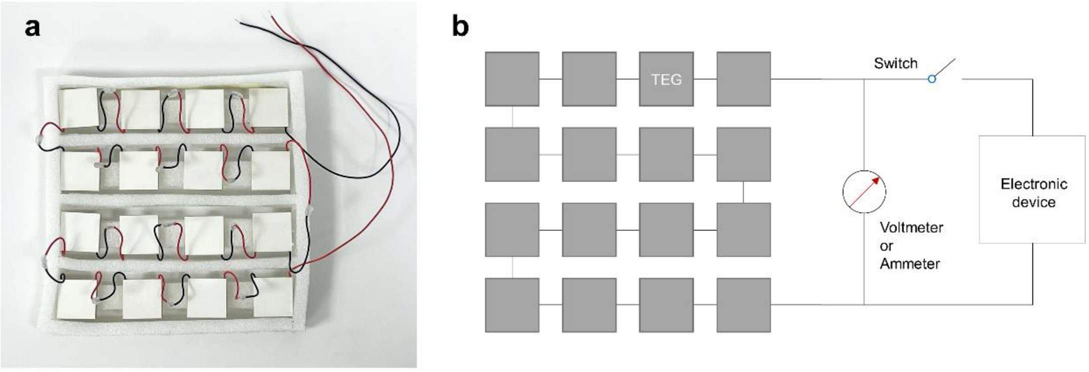

Figure S23. a) Photograph of 16 individual TEG in series. b) Circuit diagram for connecting multimeter and electric device.

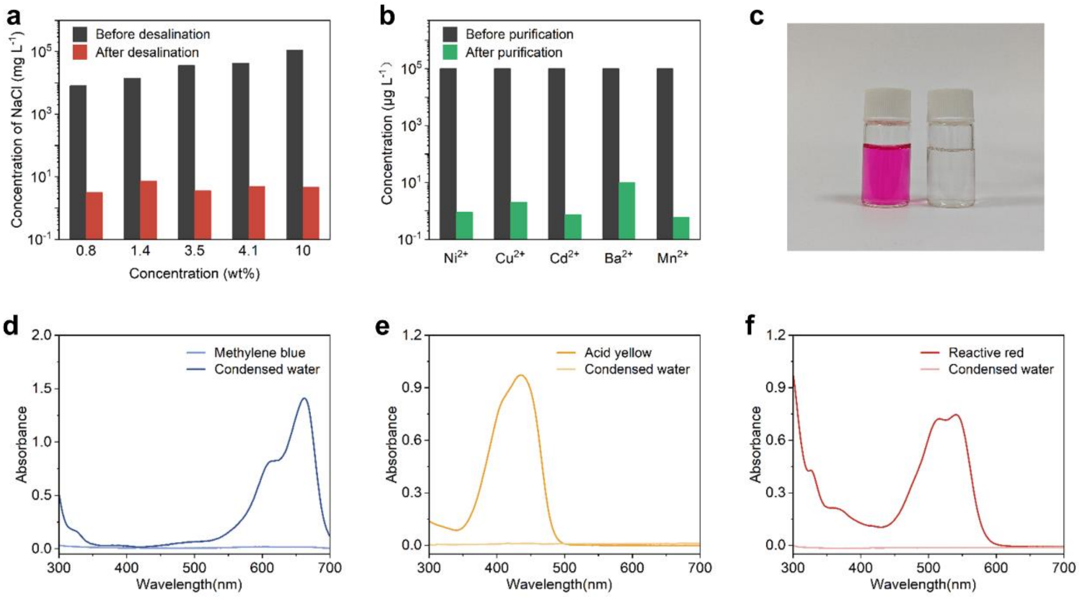

Figure S24. Purification of the CBFG system. a) NaCl concentrations of different concentrations for brine before and after solar evaporation. b) Concentrations of heavy metal ions in simulated effluents before and after solar evaporation. c) Photograph of aqueous solution of reactive red 195 before and after purification. d-f) Ultraviolet-visible absorption spectra of simulated dyeing effluents before and after solar evaporation, including (d) methylene blue, (e) acidic yellow 11, and (f) reactive red 195.

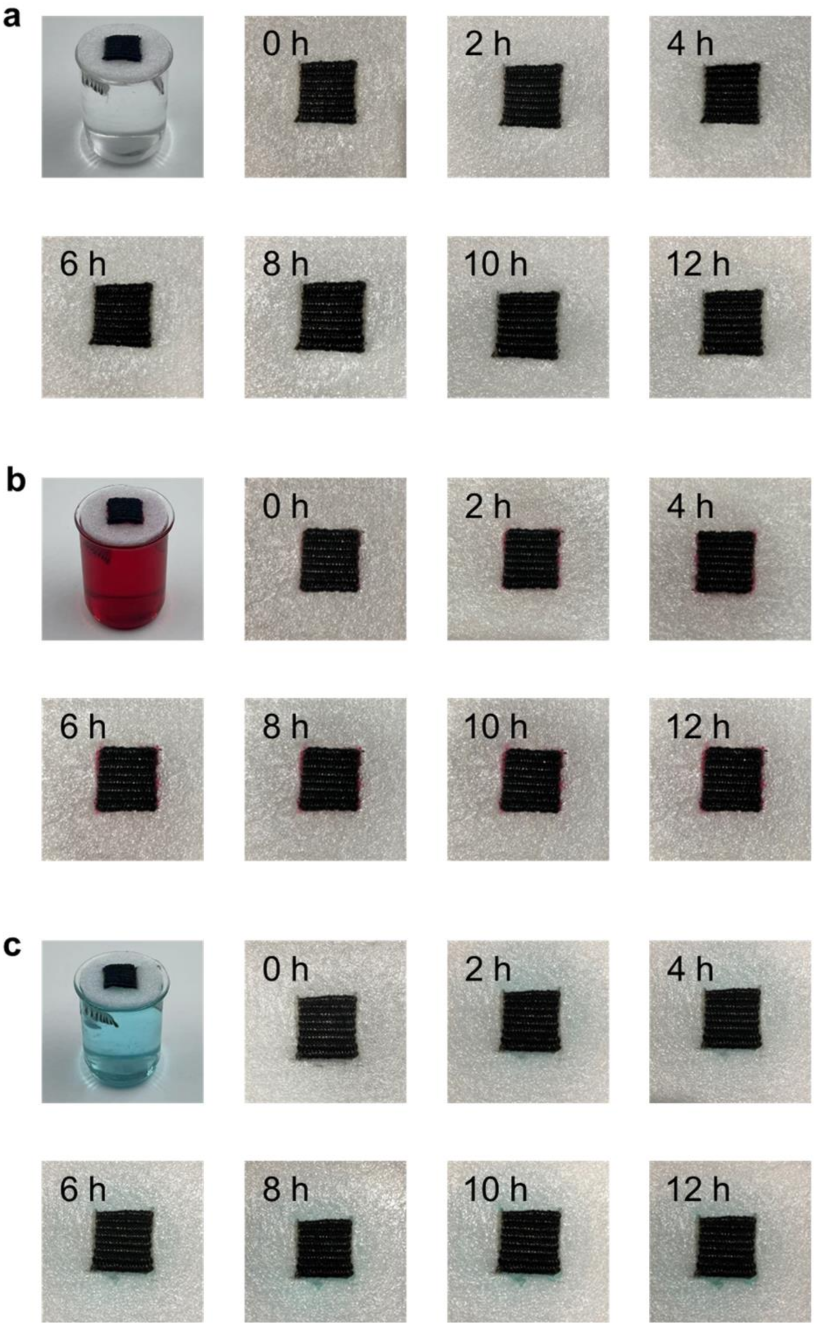

Figure S25. a) Optical photographs of evaporator surface within 12 h continuous purification with a) NaCl solution (3.5 wt%), b) reactive red dye solution (0.5 g L^{-1}), and c) $CuSO_4$ solution (10 g L^{-1}).

Note S1. Calculation of water evaporation rate

The evaporation rate (*E.R.*) was obtained from the following equation S1:

$$E.R. = \frac{\Delta m}{st} \quad \text{(equation S1)}$$

where Δm is the mass change of water evaporation, *s* denotes the evaporation area of the evaporator, *t* is the evaporation time.

Note S2. Calculation of photothermal conversion efficiency

The photothermal conversion efficiency (η) can be calculated by equation S2:

$$\eta = \frac{m h_{LV}}{P} \quad \text{(equation S2)}$$

where *m* is the difference between water evaporation in illumination and darkness, h_{LV} denotes the total enthalpy of the liquid-vapor phase transition, *P* represents the input energy of the absorber.

h_{LV} was calculated using the following equation S3:

$$h_{LV} = \alpha + \beta T + \gamma T^{1.5} + \delta T^{2.5} + \varepsilon T^{3} \quad \text{(equation S3)}$$

where *T* is the surface temperature of the CBFG at steady state under 1 kW m^{-2} solar irradiation, the values of α, β, γ, δ, ε are 2500.34, -2.2521025, -0.021465847, 3.1750136×10^{-4}, -2.8607959×10^{-5}, respectively.[2, 3]

Table S1. Water content in the top and bottom layers of fabrics with different densities.

Weaving density (warp threads×weft threads)	Water content (g)	Water content (%)
	Cotton yarn layer (bottom)	Basalt fiber layer (top)
36×260	3.07	29.7
108×500	8.52	31.5
180×800	10.61	35.6

Table S2 Comparison of water-electricity cogeneration systems.

Energy source	Materials	Evaporation rate ($kg\ m^{-2}\ h^{-1}$)	Power density ($mW\ m^{-2}$)	Ref.
Evaporation-induced	rGO/FeOOH/GO cellulose ester membrane	1.94	51.33	[4]
	CNTs film/cellulose paper	1.15	20	[5]
	MOFs/PANI/PVDF hybrid membrane	1.442	15.377	[6]
Triboelectric	Au nanoflowers/silica gel	1.356	< 0.1	[7]
Pyroelectric	Nitrogen-enriched carbon sponge	1.39	0.24	[8]
	W-doped VO_2/PVDF film	1.39	0.104	[9]
Thermo-electrochemical	PDMS/CuO/Cu polyacrylamide hydrogel	1.33	1.6	[10]
	Graphite felt	1.1	0.5	[11]
Salinity	Bio-graphene coated sponge/Nafion membrane	1.42	250	[12]
Thermoelectric	Carbonized bamboo	0.99	13	[13]
	CNT paper	0.84	240	[14]
	CNTs/cellulose nanocrystals sponge	1.36	400	[15]
	**Carbon black deposited Basalt/cotton mixed fabric**	**1.52**	**66.7**	**Our work**

References

[1] G. Xue, Y. Xu, T. Ding, J. Li, J. Yin, W. Fei, Y. Cao, J. Yu, L. Yuan, L. Gong, J. Chen, S. Deng, J. Zhou, W. Guo, *Nat. Nanotechnol.* **2017**, 12, 317.

[2] C. O. Popiel, J. Wojtkowiak, *Heat Transf. Eng.* **1998**, 19, 87.

[3] J. Wang, Q. Shi, C. Li, Y. Zhang, S. Du, J. Mao, J. Wang, *Adv. Funct. Mater.* **2022**, 32, 2201922.

[4] J. Ma, Z. Guo, X. Han, K. Guo, H. Li, P. Fang, X. Wang, J. Xin, *Carbon* **2023**, 201, 318.

[5] P. Xiao, J. He, F. Ni, C. Zhang, Y. Liang, W. Zhou, J. Gu, J. Xia, S.-W. Kuo, T. Chen, *Nano Energy* **2020**, 68, 104385.

[6] Z. Li, X. Ma, D. Chen, X. Wan, X. Wang, Z. Fang, X. Peng, *Adv. Sci.* **2021**, 8, 2004552.

[7] M. Gao, C. K. Peh, H. T. Phan, L. Zhu, G. W. Ho, *Adv. Energy Mater.* **2018**, 8, 1800711.

[8] L. Zhu, M. Gao, C. K. N. Peh, X. Wang, G. W. Ho, *Adv. Energy Mater.* **2018**, 8, 1702149.

[9] M. Jiang, Q. Shen, J. Zhang, S. An, S. Ma, P. Tao, C. Song, B. Fu, J. Wang, T. Deng, W. Shang, *Adv. Funct. Mater.* **2020**, 30, 1910481.

[10] F. L. Meng, M. Gao, T. Ding, G. Yilmaz, W. L. Ong, G. W. Ho, *Adv. Funct. Mater.* **2020**, 30, 2002867.

[11] Q. Shen, Z. Ning, B. Fu, S. Ma, Z. Wang, L. Shu, L. Zhang, X. Wang, J. Xu, P. Tao, C. Song, J. Wu, T. Deng, W. Shang, *J. Mater. Chem. A* **2019**, 7, 6514.

[12] M. Wang, Y. Wei, X. Wang, R. Li, S. Zhang, K. Wang, R. Wang, H. Chang, C. Wang, N. Ren, S.-H. Ho, *Nat. Water* **2023**, 1, 716.

[13] B. Gong, H. Yang, S. Wu, Y. Tian, X. Guo, C. Xu, W. Kuang, J. Yan, K. Cen, Z. Bo, K. Ostrikov, *Carbon* **2021**, 171, 359.

[14] Y. Duan, M. Weng, W. Zhang, Y. Qian, Z. Luo, L. Chen, *Energy Convers. Manage.* **2021**, 241, 114306.

[15] L. Zhu, T. Ding, M. Gao, C. K. N. Peh, G. W. Ho, *Adv. Energy Mater.* **2019**, 9, 1900250.
